# The Mysterious Actor—γδ T Lymphocytes in Chronic Lymphocytic Leukaemia (CLL)

**DOI:** 10.3390/cells11040661

**Published:** 2022-02-14

**Authors:** Michał K. Zarobkiewicz, Agnieszka A. Bojarska-Junak

**Affiliations:** Department of Clinical Immunology, Medical University of Lublin, 20-093 Lublin, Poland

**Keywords:** γδ T, Vδ1, Vδ2, Vδ3, CLL, chronic lymphocytic leukaemia, cytotoxicity

## Abstract

Chronic lymphocytic leukaemia (CLL) is the most common leukaemia among adults. It is the clonal expansion of B cells expressing CD19 and CD5. Despite significant progress in treatment, CLL is still incurable. γδ T cells comprise an important subset of the cytotoxic T cells. Although γδ T cells in CLL are dysfunctional, they still can possibly be used for immunotherapy. The current paper reviews our understanding of γδ T lymphocytes in CLL.

## 1. Chronic Lymphocytic Leukaemia

Chronic lymphocytic leukaemia (CLL) affects mostly older individuals, usually over 70 years old; it is also the most common leukaemia among adults in the Western world [1]. It can also affect younger adults, and the median age of diagnosis may differ between populations and regions of the world [2]. Moreover, CLL is more prevalent in males than females [3,4] and among white than black or Asian populations [4].

**Figure 1:**
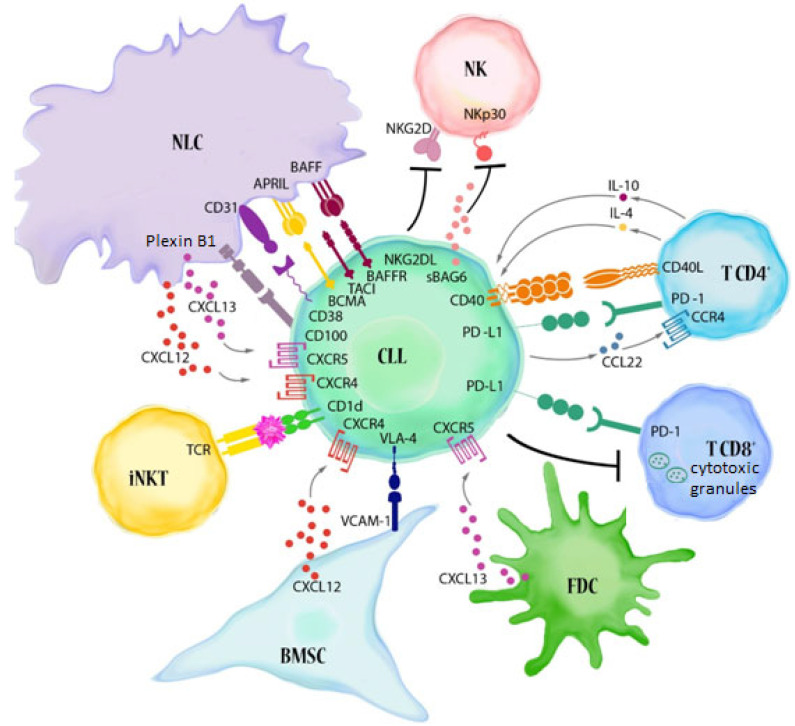
Major immune alterations in CLL. The reciprocal interactions between CLL cells and cellular elements of the immune system contribute to the building of a microenvironment that favours tumour progression. Leukaemic B lymphocytes make contact with BMSC (bone marrow stromal cells), FDC (follicular dendritic cells) and NLCs (nurse-like cells, lymphoma-associated macrophages) through adhesive molecules present on their surface (e.g., VLA-4 (CD49d)) and chemokine receptors (CXCR4 and CXCR5). These interactions, together with BCR activation, promote the survival, proliferation and migration of CLL cells. NLCs, which show phenotypic features similar to M2-like tumour-associated macrophages (TAM), express TNF family molecules: BAFF (B cell activating factor) and APRIL (A proliferation-inducing ligand) which support the survival of leukaemic B lymphocytes (BAFF binds to the BAFF-R, BCMA (B cell maturation antigen) and TACI (transmembrane activator and CAML interactor) receptors, while APRIL binds only the last two receptors) [15,22,25]. The CD31 molecules present on NLCs are ligands for the CD38 found on leukaemic B lymphocytes. Their interaction induces proliferation and prolongs the survival of CD38-positive lymphocytes [15]. A similar effect is caused by the interaction of plexin 1 with the CD100 present on CLL cells. NLCs share the ability to express plexin B1 with BMSC, FDC and activated T lymphocytes [26]. The interaction of CD40 with CD154 (CD40L) on T cells, and the IL-4 released by them, promotes the inhibition of apoptosis in leukaemic cells. Moreover, CD4+ and CD8+ T cells display high levels of exhaustion markers, including PD-1. CLL cells express high levels of PD-L1. The PD-1/PD-L1 axis favours the immune evasion of CLL cells from T cell cytotoxicity [15,27]. Several factors also contribute to reduced NK cell cytotoxicity, including the low expression of NK cell-activating receptors, such as NKp30. Moreover, soluble NKG2D ligands and soluble BAG6 (BAG cochaperone 6) can be released by CLL cells [25]. Another important element of the CLL microenvironment, namely, invariant NKT (iNKT) cells, can directly recognize the antigens presented by neoplastic lymphocytes and lead to their destruction. iNKT cells have the ability to activate and expand in response to the antigens presented by CD1d [14].

CLL results from the clonal expansion of abnormal B cells that co-express B cell markers (CD19, CD20) with T cell-specific proteins (CD5) [5]. CLL belongs to the group of indolent lymphomas, and usually does not have a very aggressive course and has a relatively long overall survival time [6]. There is no clear benefit of early treatment; thus, a wait-and-watch strategy is usually followed [7]. Indeed, less than half of the patients require treatment shortly after diagnosis [8]. Nevertheless, this disease is highly heterogeneous, with some patients having a more aggressive course, particularly those with unmutated immunoglobulin heavy chain genes (IGHV), and del(17p), del(11q) and TP53 gene mutations [9,10]. Immunophenotypic markers, such as CD38 and ZAP-70, are widely accepted as indicators of a poor prognosis [11,12]. CD38 expression correlates with IGHV mutational status, but may also have independent prognostic significance. ZAP-70 is an intracellular protein that is normally expressed in T cells, but is aberrantly expressed in CLL cells in a subset of patients. ZAP-70 expression may be a strong independent predictor of poor prognosis. High ZAP-70 expression in leukaemic B cells correlates with unmutated IGHV status [13]. It is worth noting that, in addition to the intrinsic characteristics of the malignant clone, the main causes of CLL progression are profound defects in the immune system and the ability of leukaemic cells to bypass recognition and elimination. Neoplastic cells and cellular components of the microenvironment are interrelated and co-evolve, shaping each other in the course of the disease [14]. Key elements of the microenvironment are monocyte-derived nurse-like cells (NLCs), mesenchymal stromal cells, T cells, NKT and NK cells, which communicate with CLL cells through a complex network of adhesion molecules, chemokine receptors, tumour necrosis factor (TNF) family members and soluble factors (Figure 1) [15]. CLL cells also promote the expansion and recruitment of immunosuppressive cells, including myeloid suppressor cells (MDSC) and T regulatory (Treg) cells, in order to escape from the control of the immune system [16,17]. Intriguingly, CLL clones often have features of regulatory B (Breg) cells. Breg and leukaemic B cells share phenotypic characteristics, both express CD5, CD24 and CD27, and both have low surface IgM levels. They also share physiological analogies (such as IL-10 production), suggesting that CLL B cells might negatively control T cell activation and immune response [18,19]. Interactions between CD40-expressing leukaemic B cells and CD40 ligands (CD40L) on activated CD4+ T cells promotes the proliferation of CLL cells and the upregulation of anti-apoptotic proteins. Moreover, T cells provide pro-survival signals through soluble factors, such as interleukin-4 (IL-4) and interferon-gamma (IFN-γ), which upregulate anti-apoptotic Bcl-2 in CLL cells [20,21]. The T cell number is increased in the peripheral blood of CLL patients; in particular, the CD8+ T cell count rises, causing a decline in the CD4:CD8 ratio. Despite their increased numbers, T cells show profound functional defects [22]. Both CD4+ and CD8+ T cell subpopulations display functional defects, including impaired immune synapse formation with antigen-presenting cells, impaired cytokine production, degranulation and antitumor cytotoxicity [23]. Furthermore, T cells in CLL exhibit markers of chronic activation and exhaustion, such as PD-1, contributing to inhibited effector function and impaired immunological synapse formation [23,24].

Nowadays, CLL is still an incurable disease. Clinical resistance may occur both through the primary biological features of malignant cells or through resistance, which arises from the crosstalk with the surrounding tumour microenvironment. Studies defining the importance of the CLL microenvironment and BCR signalling have resulted in the development of drugs, such as ibrutinib (a Bruton’s tyrosine kinase (BTK) inhibitor) [28]. Simultaneously, an examination of the mechanisms that promote the survival of CLL cells has led to the development of venetoclax (a Bcl-2 inhibitor) [29]. Despite the success of these agents, challenges persist. Novel immunotherapeutic strategies, such as chimeric antigen receptor (CAR)-transduced T cells and immune checkpoint blockades, have shown discouraging results in CLL [30,31,32], mainly due to defects in the effector T cells [33]. Therefore, it is required to study the therapeutic potential of other effector cells in order to find more effective immunotherapeutic strategies.

## 2. γδ T Cells in Human

γδ T cells are a subset of T cells, comprising approximately 2–5% of total T lymphocytes in the peripheral blood. Human γδ T cells incorporate one of three δ chains (δ1, δ2 or δ3) and one of six γ chains (γ2, γ3, γ4, γ5, γ8 or γ9) [34]. γδ T cells in the peripheral blood are mostly divided into subsets based on the δ chain that they use, namely, Vδ1, Vδ2 and Vδ3. Vδ1 subset respond to self-antigens, such as MICA, MICB and ULBPs, which are frequently upregulated in cancers; Vδ2 respond to so-called phosphoantigens [35]. These phosphoantigens can be of bacterial (e.g., HMB-PP) or eukaryotic origin (isopentenyl pyrophosphate, IPP) [36,37]. Vδ3 cells are far less understood than either Vδ1 or Vδ2; Vδ3 cells comprise a significant population of hepatic γδ T cells, but in many individuals, they are virtually absent from the peripheral blood [38]. They may, however, be significantly expanded in the peripheral blood in the course of some viral infections and other diseases, e.g., systemic lupus erythematosus [39,40,41]. Vδ3 lymphocytes can recognise the glycolipids presented in the context of CD1d [39].

γδ T cells are involved in autoimmune diseases [42], asthma [43] and infection surveillance [44,45]. Both Vδ1 and Vδ2 subsets express high cytotoxic potential and are important in cancer immunosurveillance [35,46]. The tumour micro-environment can also promote regulatory functions in γδ T cells, simultaneously lowering their cytotoxic potential [47].

## 3. γδ T Recognition of Tumour Cells

In contrast to ɑβ T cells, γδ T lymphocytes recognise tumour cells independently of HLA restrictions [48]. γδ T cells express a variety of recognition receptors apart from their TCR-KG2D is uniformly expressed by all three major subsets, namely, Vδ1, Vδ2 and Vδ3 [49]. Knowledge about the recognition/activation receptors on cells in Vδ3 is currently limited. Cells in Vδ1 and Vδ2 express similar receptors and transmembrane proteins, including TRAIL, FasL, 2B4, DNAM-1 and NCRs [50]. NCRs include NKp30, NKp44 and NKp46 [51]. NKp44 recognises several ligands, including HSPGs (heparan sulfate proteoglycans) and MLL5 isoform (21spe-MLL5), that can be overexpressed on the surface of cancerous cells [52]. B7-H6 is one of the major ligands for NKp30, while HSPGs are major cancer-derived ligands for NKp46 [53]. The major ligand for DNAM-1 is CD155, also known as PVR [54]. It seems that cells in Vδ3 often express NKG2D, but rarely or never NKG2C and NKG2A [39].

Vδ2 cells are potently activated by phosphoantigens, including isopentenyl IPP. IPP is generated in the mevalonate pathway of cholesterol synthesis in eukaryotic cells. Under physiological conditions, the amount of IPP generated by healthy tissue is too low for γδ T activation. However, the mevalonate pathway is dysregulated in numerous cancers; thus, they accumulate sufficient IPP amounts to potently activate Vδ2 cells [48]. Phosphoantigens are not recognised directly; rather, they merely cause conformational changes in butyrophilins 2A1 and 3A1 (BTN2A1 and BTN3A1), which can later be sensed by γδ T cells via TCR [36,37,55,56]. Additionally, Vδ2 and Vδ1 cells express CD16 (FcγRIII), and their cytotoxic response against certain cancers can be significantly enhanced by therapeutic monoclonal antibodies [57,58,59]. TCR-dependent antigen recognition by non-Vδ2 γδ T cells in humans is poorly understood, although a range of antigens (mostly self-antigens) have been associated with certain γδ TCRs [60]. Still, the exact mode is not clear, e.g., Vδ1 cells recognise both empty CD1 molecules, as well as those presenting glycolipid or phospholipid, although in the latter case the affinity is higher [60,61]. Some authors even suggest that IgG antibodies may be considered to be yet another ligand for γδ TCR [62]. For a wider overview of the topic, we suggest a recent review article by Malte Deseke and Immo Prinz [60].

**Figure 2:**
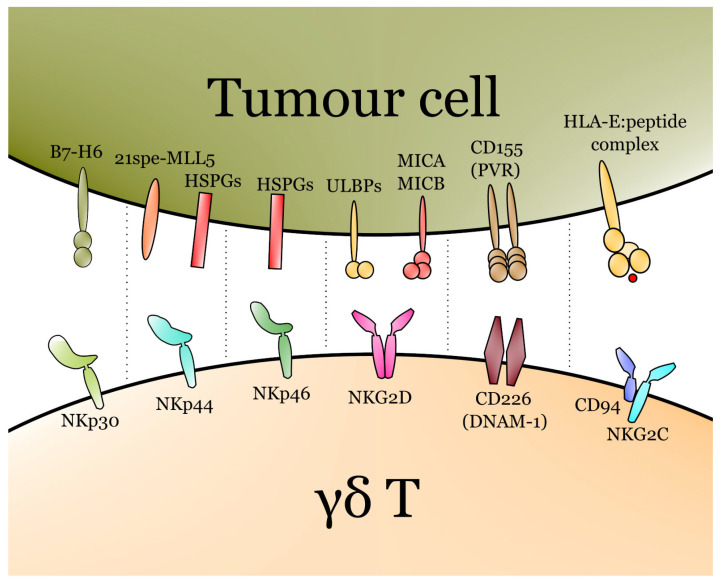
Major activating receptors of γδ T cells with their respective ligands. The T cell receptor (TCR) also plays a major role in the activation of γδ T, though it is not depicted due to the complexity of TCR–ligand interactions in γδ T cells and differences between Vδ1, Vδ2 and Vδ3.

Thus, γδ T cells can be activated either directly via TCR or via one of the numerous activating receptors. Moreover, the mode of activation depends highly on the type of cancer: two types of signal, e.g., TCR + NKG2D, may be necessary in some cases [63]. Major activating receptors with their ligands are summarised in Figure 2.

Similar to αβ T, γδ T cells can express a plethora of inhibitory receptors and checkpoint molecules. γδ T lymphocytes express PD-1, and the blockage of the PD-1-PD-L1(L2) axis increases their cytotoxic potential [64,65]. Moreover, PD-1 is rapidly upregulated on Vδ2 cells following phosphoantigen activation [65]. Interestingly, PD-1+ Vδ2 cells are capable of IL-2 production [65]. Human γδ T cells also express BTLA [66], which negatively regulates γδ T proliferation in response to phosphoantigens [67]. Finally, γδ T lymphocytes may have TIM-3, TIGIT and LAG-3 on their surface [68,69], but only rarely do they have CTLA-4 [71, our own unpublished data]. Indeed, TIM-3 is highly expressed on γδ T cells in certain advanced cancers and negatively correlates with the cytotoxic potential of γδ T cells [70]. This effect is mediated by the downregulation of both perforin and granzyme B [70], and also by the lower production of IFN-γ and TNF [71]. High TIGIT expression on γδ T lymphocytes correlates with poor responses to chemotherapy and lower overall survival in acute myeloid leukaemia [69]. Apart from classical checkpoint molecules, γδ T cells may express a variety of inhibitory KIRs (killer Ig-like receptors), e.g., KIR3DL or KIR2DL, as well as an inhibitory member of the NKG2 family, namely NKG2A [72].

## 4. Vδ1 γδ T Cells Are Expanded in the Peripheral Blood of CLL Patients

γδ T cells are capable of rapidly responding to tumours, along with significant expansion [73]. Indeed, γδ T cells in CLL are significantly expanded, both in terms of absolute count, as well as with their percentage among T cells [74]. This can be attributed mostly to the proliferation of Vδ1 γδ T cells [74,75,76]. Although no correlation between γδ T percentage or count and the clinical course of CLL was observed [74,77], the usage of Vδ1 tends to rise [75,78] and Vδ2 decreases with the progression of CLL [75]. Indeed, de Weerdt observed a non-significant decrease in the percentage of Vδ2 cells in the peripheral blood of CLL patients, as well as a tendency for an increase in the absolute count [79]. On the other hand, only patients with low CD38 expression and mutated IGVH tend to have a rise in the Vδ1 subset, and those with unfavourable prognostic factors seem to have a very low count of Vδ1 cells [76]. Własiuk et al. observed no difference in total γδ T percentage between ZAP-70-positive and -negative patients, but noticed a lower percentage in CD38+ cases [77]. γδ T cells are frequently expanded also in other cancers, e.g., within the tumour in breast cancer or rectal cancer; in those cases, this expansion may have a negative prognostic value, and seems to significantly increase with the disease progression [80,81]. Although it appears that the opposite is true for CLL, this hypothesis requires further testing. Moreover, both Vδ1 and Vδ2 cells can have regulatory phenotypes, thus promoting immunosuppression [79,82], which did not gain significant attention in CLL.

Usually around one-quarter of human γδ T cells in the peripheral blood express CD8 [83]. CD8+ γδ T cells from bone marrow recipients seem to have an effector phenotype, with a higher capacity for cytokine production and cytotoxicity than their CD8- counterparts [84]. In fact, more than half of γδ T cells in CLL weakly express CD8 [74]. An investigation into whether a higher percentage or count of CD8+ γδ T cells may have some prognostic value in CLL is still required.

## 5. Vδ1 Cells Are More Cytotoxic towards CLL Clones Than Vδ2

γδ T lymphocytes have potential prognostic value in various human cancers [85,86]. Although there is no hard evidence that this is also the case in CLL, there are several observations suggesting it. First of all, patients with a high Vδ1 count usually have a more stable disease and a lower risk of progression [76]. Vδ1 cells from CLL patients usually have a cytotoxic profile, which is manifested by higher granzyme B expression compared to controls [78]. Still, as those cells are CD27-negative, they may be functionally exhausted. The CD27-negative compartment can be further subdivided into effector memory, terminally differentiated effector memory and exhausted γδ T cells [87]. In fact, Vδ1 cells from low-risk CLL patients tend to proliferate and express IFN-γ and TNF in response to autologous leukaemic B cells [76]. At the same time, Vδ2 cells express very low IFN-γ and TNF in response to autologous leukaemic B cells [76]. Moreover, CLL-derived Vδ2 cells produce lower amounts of both TNF and IFN-γ compared to healthy-derived cells [88]. Vδ1 cells also have higher NKG2D expression than Vδ2 cells in CLL; neither exert, however, any spontaneous cytotoxicity against autologous leukaemic B cells [76]. Vδ1 cells activated with polyclonal mitogen are, on the other hand, relatively good killers of autologous leukaemic cells [76]. Furthermore, this cytotoxicity is exerted against ULBP-expressing B-CLL clones, and is completely blocked with anti-NKG2D antibodies [76]. Thus, it seems that Vδ1 γδ T cells are better responders to CLL cells than Vδ2. Indeed, Vδ1 cells have been previously proposed to be superior to Vδ2 cells for immunotherapy [89]; Vδ1 cells are also more cytotoxic against adherent cells than Vδ2 cells in in vitro cytotoxicity [90]. In vitro-expanded Vδ1 showed very high cytotoxicity against multiple myeloma cells, irrespective of whether they originated from the patients’ or healthy volunteers’ blood [91].

## 6. Vδ1 Cells for the Cellular Immunotherapy of CLL

Both Vδ1 and Vδ2 cells can potentially be used for cellular immunotherapy. Vδ1 cells seem to be a better option, as they are less affected by CLL burden and show higher overall cytotoxic potential against CLL clones. Indeed, Correia et al. showed the superiority of pan γδ T cells expanded with general T cell mitogen PHA instead of Vδ2-specific HMBPP in the lysis of CLL leukaemic cells [92]. While HMBPP promoted the expansion of Vδ2 cells only, PHA stimulated Vδ1 cell proliferation to a higher extent [92]. The superior cytotoxicity could be attributed to higher NKp30 and NKp44 expression by Vδ1 cells [92]. Almeida et al. proposed a two-step clinical-grade protocol for Vδ1 cell expansion from the peripheral blood of either healthy donors or CLL patients, yielding up to a 2500-fold increase in Vδ1 cell numbers [93]. Interestingly, IL-4 is used along with IFN-γ during the first 14 days of culture; after this phase, cells have a relatively low expression of NKp30, NKp44 and NKG2D, a receptor crucial for their cytotoxic activity [93]. Thus, for the second step of expansion, IL-15 is used along with IFN-γ to promote cytotoxic potential [93]. Indeed, such expanded Vδ1 cells show high cytotoxicity against both primary CLL cells (autologous or allogeneic) and EBV-positive CLL line MEC-1 [93]. NKp30 and NKp44 seem to be crucial for cytotoxicity against CLL cells—the combined blockage of those two receptors completely eliminates cytotoxicity [93].

Although Vδ2 cells are far easier to expand in vitro for clinical use, there are some new and interesting protocols for clinical-grade expansion of Vδ1 cells [93,94]. Moreover, in vitro-expanded pan-γδ T cells with the prevalence of Vδ1 cells seem to be even more promising for immunotherapy. They effectively kill various leukaemic cell lines at a low effector:target ratio, and have a predominantly cytotoxic immunophenotype with a very low expression of checkpoint molecules [95].

## 7. Vδ2 in CLL Patients Are Dysfunctional

As mentioned, Vδ2 cells are less cytotoxic against CLL clones than Vδ1. Even Vδ2 cells from healthy donors fail to demonstrate cytotoxicity against CLL leukaemic cells, but significant cytotoxicity can be exerted by the addition of an anti-CD20 antibody (rituximab) [57]. On the other hand, both Vδ1 and Vδ2 cells from healthy donors seem to be cytotoxic against EBV-infected CLL-line MEC-1 without any additional stimulus; Vδ1 cells are also cytotoxic against EBV-negative CLL line TMD2 (Vδ2 cells were not tested) [96]. Moreover, de Weerdt et al. reported the activation of Vδ2 cells by CLL cells, as well as significant granzyme B-mediated cytotoxicity against them [88]. When Vδ2 cells were derived from CLL patients there was, however, only very little cytotoxicity [88]. Moreover, CLL-derived Vδ2 cells tend to be more differentiated and to express less granzyme B than those from healthy volunteers [88,97].

The difference in the cytotoxic potential of CLL- and healthy volunteer-derived Vδ2 cells may be related to the difference in the expression of co-inhibitory molecules. Indeed, although no difference in LAG-3 and PD-1 was observed, a significant upregulation of CD160, another co-inhibitory molecule, was noted in CLL patients [88]. It seems that the dysfunction of Vδ2 cells is mediated through CLL leukaemic cells, as the co-culture of healthy volunteer-derived Vδ2 cells with CLL cells promotes similar dysfunction [88]. Interestingly, it seems that those changes are reversible—the in vitro expansion or activation of CLL-derived Vδ2 cells restores their properties; their cytokine production and degranulation, as well as their cytotoxicity, are comparable to that of healthy ones [88]. Furthermore, RNAseq revealed the upregulation of around 100 genes and the downregulation of another 500 in CLL-derived γδ T cells compared to healthy subjects [88]. In vitro activation seems to significantly alleviate those changes [88]. Thus, it looks like the CLL cells or general tumour microenvironment of CLL promotes the significant dysfunction of Vδ2 cells. Dysfunctional Vδ2 lymphocytes have been observed in various parasitic or viral infections, as well as in cancer patients [98,99,100,101]. Moreover, in older adults with cancer, this dysfunction overlaps with signs of senescence [98]. A general decrease in the γδ T percentage and count was observed in older subjects, as was a weaker response to the phosphoantigen stimulation of Vδ2 cells [102,103]. Moreover, γδ T cells from older subjects show higher basal activation, and are also more prone to undergo apoptosis [103,104]. Those changes are at least partially related to cytomegalovirus (CMV) infection [105]. Indeed, a most striking decrease in Vδ2 count can be observed in older CMV-positive individuals [106]. Moreover, an increase in Vδ1 can also be noted [106]. Thus, it seems that the changes observed in CLL should be partially attributed to senescence. Nevertheless, control groups in CLL studies usually have a similar age and sex structure to CLL patients; therefore, one can conclude that the observed differences are mostly related to the disease, and only partially to the age. Finally, a different disease course and shorter survival were noted among CMV+ CLL patients [107].

## 8. Poor response of Vδ2 to Phosphoantigen Stimulation Has a Negative Prognostic Value in CLL

Zoledronate and phosphoantigens, such as IPP or HMBPP, are frequently used to expand Vδ2 cells in vitro, or for the assessment of proliferation capabilities [108]. Zoledronate was also tested for in vivo expansion in various human cancers [109]. Their proliferation capability was also tested in CLL patients. γδ T from CLL patients proliferate to various extents after the zoledronate stimulation of PBMCs [88,97]—based on their response, Coscia et al. divided CLL patients into responders and low responders [97]. Interestingly, low responders tended to have a significantly higher absolute count of Vδ2 cells, while responders had a significantly higher expression of NKG2D on Vδ2 cells [97]. It is currently not clear what is the underlying difference between responders and low responders. Although the expression of CD6, one of the activatory T cell receptors [110], did not differ between responders and low responders, the expression of its ligand (CD166) was significantly higher on CLL leukaemic cells from responders [97]. Although responders and low responders do not differ in terms of ZAP-70 and CD38 expression, cytogenetic abnormalities, β2-microglobulin or LDH levels, they differed significantly in IGVH mutational status—only 20% of responders and 51% of low responders had unmutated IGVH [97]. Moreover, IGVH-mutated subjects tend to have higher NKp44 expression on γδ T than unmutated ones [97]. On the other hand, IGVH mutational status seems to have no importance on cytotoxic potential and the degranulation of Vδ2 cells [88]. The importance of IGVH mutation can possibly be attributed to the activity of the mevalonate pathway in CLL cells; cases with unmutated IGVH have a higher level of activity [97]. Despite being initially counter-intuitive, this can be explained by the functional exhaustion of Vδ2 cells by constant stimulation with IPP in patients with unmutated IGVH; indeed, a higher initial Vδ2 count is associated with a shorter time to first treatment [97]. The exhaustion mechanism is also supported by studies with the in vivo application of aminobisphosphonates; initially, the activation and proliferation of Vδ2 cells are observed but, after prolonged treatment, Vδ2 count and responsiveness to phosphoantigens decrease significantly [111,112]. Low responders also have significantly higher amounts of T regulatory cells [97]. Finally, responders have a significantly longer time to first treatment than low responders [97].

## 9. Effect of Ibrutinib on γδ T Cells

Ibrutinib is a BTK inhibitor; BTK is a crucial kinase for BCR signalling [113]. It is one of the most effective drugs, with a response rate over 90% in clinical trials [114]. Although ibrutinib should not act on cells other than B lymphocytes, it shows potential off-target effects on other kinases as well [115]. Moreover, BTK may also play a limited role in T cell biology [116]. Thus, it is important to understand ibrutinib’s impact not only on B and ɑβT, but also on γδ T cell biology.

Preliminary data suggest the important influence of ibrutinib on the function of γδ T cells. Ibrutinib promotes TNF production and cytotoxicity, and also decreases IL-4 production in CLL-derived Vδ2 cells [88]. On the other hand, Risnik et al. reported a significant decrease in TNF and IFN-γ production by healthy donor-derived Vδ2 cells stimulated with HMBPP and ibrutinib, and no difference was observed for CLL-derived cells [117]. Ibrutinib seems to also negatively affect the activation and degranulation potential of Vδ2 cells from both CLL patients and healthy donors [117]. Importantly, ibrutinib may reduce γδ T response against M. tuberculosis, thus raising the potential risk for life-threatening infections [118]. Ibrutinib and other BTK inhibitors are relatively widely used for the therapy of various haematological malignancies [119]. Thus, it is important to further our understanding of ibrutinib’s impact on γδ T fitness.

## 10. Perspectives for the Use of γδ T Cells in CLL Immunotherapy

There are currently more than 10 companies that are working on the clinical uses of γδ T cells in cancer immunotherapy [48]. They utilise different approaches, from the in vivo activation of γδ T cells through off-the-shelf in vitro-expanded allogenic Vδ2 γδ T cells to CAR γδ T cells [48]. It is technically possible to use allogenic γδ T cells, as they should not attack the healthy cells of the recipient. Indeed, a recent clinical study proved the safety and limited efficacy of such in vitro-expanded allogenic Vδ2 γδ T [120]. The in vitro expansion of Vδ2 cells from CLL donors has severely limited potential; thus, the motion to employ allogenic ones looks particularly promising. It offers a chance to overcome the exhaustion of Vδ2 cells in CLL patients. Using DOT (delta-one-T cells) can be a similarly promising approach, in which autologous γδ T cells can be effectively expanded in vitro, with a predominance of Vδ1 cells in the final product [93]. This approach still requires clinical trial to assess both safety and efficacy.

The efficacy of both “own” circulating and in vitro-expanded γδ T cells can potentially be significantly increased with appropriate drugs, e.g., double specific antibodies [48,88,120]. Moreover, γδ T cells can be used for the development of CAR T cells. In fact, γδ T CAR T cells offer a higher potential than αβ ones, as γδ T recognise tumour cells, even when the target protein of CAR is downregulated [121]. Moreover, the efficacy of γδ T-based CAR-T cells can potentially be further increased by additional drugs, such as the previously mentioned double specific antibodies or zoledronate [48,121]. Nevertheless, there are still significant challenges that have to be overcome, e.g., choosing the best method for in vitro expansion (i.e., one that offers a very high yield of highly active γδ T cells). Current approaches range from a simple zoledronate + IL-2 regimen through PHA stimulation to a complex approach with numerous cytokines used, as proposed by Almeida et al. [48,49,93].

γδ T-based immunotherapy is a promising option for the treatment of CLL. Still, there are numerous obstacles that have to be overcome before their potential can be widely used in a clinical setting.

## 11. Conclusions

The importance of γδ T cells in CLL is not fully understood. However, it seems that phosphoantigen-reactive Vδ2 cells tend to be overstimulated and exhausted, while Vδ1 cells seem to be generally more responsive to CLL clones. Thus, although Vδ2 cells may not be fit for the cellular immunotherapy of CLL, the Vδ1 cells look promising.

## Abbreviations

2B4	natural killer cell receptor 2B4: CD244
BCR	B cell receptor
CAR	chimeric antigen receptor
CTLA-4	cytotoxic T lymphocyte-associated protein 4
DNAM-1	DNAX accessory molecule-1
EBV	Epstein–Barr virus
FasL	Fas ligand
HMB-PP	(E)-4-hydroxy-3-methyl-but-2-enyl pyrophosphate
KIR2DL	killer cell immunoglobulin-like receptor 2DL
LAG-3	lymphocyte activation gene 3
LDH	lactate dehydrogenase
MICA	MHC class I chain-related protein A
MICB	MHC class I chain-related protein B
MLL5	mixed lineage leukaemia 5
NCRs	natural cytotoxicity receptors
NKG2A	natural killer group 2A
NKG2C	natural killer group 2C
NKG2D	natural killer group 2D
NKp30	natural cytotoxicity triggering receptor 3
NKp44	natural cytotoxicity triggering receptor 2
PHA	phytohaemagglutinin
PD-1	programmed cell death protein 1
PD-L1	programmed death-ligand 1
PVR	polio virus receptor
TCR	T cell receptor
TIGIT	T cell immunoreceptor with Ig and ITIM domains
TIM-3	T cell immunoglobulin and mucin domain-containing-3
TRAIL	TNF-related apoptosis-inducing ligand
ULBPs	UL16-binding proteins
ZAP-70	zeta-chain-associated protein kinase 70
